# A Tailored Enhanced Recovery After Surgery (ERAS) Approach for Acute Pain Management in Elective Cesarean Deliveries: A Randomized Controlled Trial

**DOI:** 10.7759/cureus.82129

**Published:** 2025-04-12

**Authors:** Sankarnarayanan Rajendran, Shivanand L Karigar, Shreedevi Kori, Santosh Alalamath, Pratibha S D

**Affiliations:** 1 Department of Anesthesiology, Shri BM Patil Medical College Hospital and Research Centre, BLDE (Deemed to be University), Vijayapura, IND; 2 Department of Obstetrics and Gynecology, Shri BM Patil Medical College Hospital and Research Centre, BLDE (Deemed to be University), Vijayapura, IND

**Keywords:** cesarean delivery, enhanced recovery after surgery (eras), intraoperative warming, maternal satisfaction, postoperative analgesia

## Abstract

Background

Cesarean delivery is a significant milestone in a mother’s life, often marked by joy. However, the postoperative period can be physically challenging, especially for those undergoing the procedure due to maternal or fetal indications.

Objectives

This study aimed to evaluate the effectiveness of a tailored enhanced recovery after surgery (ERAS) protocol in managing acute pain following elective cesarean deliveries. Traditional protocols often prolong maternal discomfort and delay recovery.

Methods

Eligible patients who provided written informed consent were randomized into two groups: the ERAS protocol (EP) group and the routine protocol (RP) group. Both groups underwent comprehensive preanesthetic evaluations, including history-taking, systemic examination, airway assessment, and standard blood investigations. Patients in the EP group were allowed to consume clear fluids up to two hours before surgery, whereas those in the RP group adhered to the conventional six-hour fasting protocol.

Results

Each group included 50 patients. Intraoperative shivering was significantly lower in the EP group (22.7%; five patients) compared to the RP group (77.3%; 17 patients) (p < 0.005). Hypotension occurred in 10 EP patients versus 32 in the RP group (p < 0.005). At 24 hours post-surgery, pain scores measured using the Visual Analogue Scale were significantly lower in the EP group both at rest (1.76 ± 0.82 vs. 2.96 ± 0.95) and during movement (2.46 ± 0.81 vs. 3.78 ± 0.89) (p < 0.005 for both). Opioid use was also markedly reduced in the EP group, with only two patients (4%) requiring opioids postoperatively, compared to 25 patients (50%) in the RP group (p < 0.005).

Conclusions

The tailored ERAS protocol significantly improved acute postoperative pain management in cesarean deliveries. Patients in the ERAS group experienced fewer intraoperative complications, reduced opioid consumption, faster mobilization, shorter hospital stays, and higher overall satisfaction.

## Introduction

Spinal anesthesia is highly effective for managing intraoperative pain; however, its analgesic effect typically lasts for only about 140 minutes [1]. While some level of pain is expected as part of the normal postoperative healing process, it is often poorly controlled, leading to negative consequences. Inadequate postoperative pain management can contribute to the progression from acute to chronic pain [2]. Studies have shown that the prevalence of moderate to severe postoperative pain within the first 24 hours after cesarean delivery is as high as 85.5% (95% CI: 81.4-89.0%) [3].

Managing acute pain after cesarean delivery presents unique challenges for anesthesiologists. These patients are typically motivated to remain awake, comfortable, and mobile to care for their newborns. Although opioids remain a key component of multimodal analgesia, their use is associated with side effects such as nausea, vomiting, pruritus, sedation, and respiratory depression - especially at higher doses. Therefore, strategies aimed at minimizing opioid use are particularly beneficial in this population.

Over the past decade, several protocols and guidelines have been introduced to support enhanced recovery after surgery (ERAS). The ERAS guidelines specific to cesarean delivery provide comprehensive perioperative recommendations with a strong focus on maternal care [4-6]. However, acute pain management is not a primary focus of these guidelines. To address this gap, our medical center developed a tailored ERAS protocol that builds upon existing guidelines and incorporates clinical experience, with a greater emphasis on optimizing acute pain control.

As part of this approach, we enhanced the ERAS protocol by incorporating an ultrasound-guided bilateral transversus abdominis plane (TAP) block for postoperative pain relief. The use of ropivacaine in the TAP block has been shown to significantly reduce postoperative pain scores on the Visual Analogue Scale (VAS) compared to placebo. Additionally, patients who received the TAP block required substantially less morphine during the first 48 hours post-surgery (18 ± 14 mg vs. 66 ± 26 mg; p < 0.001), with consistently lower consumption recorded at 12-hour intervals up to 36 hours postoperatively [7].

## Materials and methods

Study design and setting

This randomized controlled trial was conducted in the Department of Anesthesiology at Shri BM Patil Medical College Hospital and Research Centre, BLDE (Deemed to be University) in Vijayapura, India. The study received ethical approval from the BLDE (Deemed to be University) Ethical Committee on April 10, 2023. It was also registered in the Clinical Trials Registry - India (CTRI) under the registration number CTRI/2024/07/070886. The trial was conducted over a six-month period, from July 2024 to February 2025.

Sample size and randomization

A previous study by Pan et al. reported the mean ± SD of postoperative pain scores at 24 hours to be 3.50 ± 1.76 in the control group and 2.46 ± 1.58 in the ERAS group, respectively [8]. Based on these values, a minimum of 50 participants per group (total sample size of 100, with equal allocation) was calculated to achieve a power of 90% and a two-sided significance level of 5% to detect a true difference in means between the groups.

The sample size formula used was:

\[ N = 2\left(\frac{(Z_{\alpha} + Z_{\beta}) \cdot S}{d}\right)^2, \]

where Zα is the critical value of the normal distribution at α (confidence level, 95%), Zβ is the critical value of the normal distribution at β (power, 90%), S is the common SD, and d is the clinically significant difference between the two means.

Study population

Eligible participants were parturients aged 25 to 45 years, with a gestational age of 38 weeks, scheduled for elective cesarean delivery under spinal anesthesia. All participants were required to provide informed consent and have no history of significant cardiovascular, coagulation, or metabolic disorders.

Inclusion Criteria

Participants included parturients scheduled for planned cesarean delivery who were classified as American Society of Anesthesiologists Physical Status grade II and were between 25 and 45 years of age.

Exclusion Criteria

Individuals were excluded if they had hypotension/hypertension, obesity, fetal compromise, preterm gestation, coagulopathies, were younger than 25 years, had allergies to study agents, or had contraindications to the TAP block.

Methodology

Preanesthetic Evaluation

All enrolled patients underwent a comprehensive preoperative assessment that included a detailed medical history and physical examination. The history addressed underlying medical conditions, prior surgeries, anesthesia exposures, and previous hospitalizations. The physical examination evaluated overall health by measuring vital signs (heart rate, blood pressure, respiratory rate, height, and weight) and assessing the cardiovascular, respiratory, central nervous, and vertebral systems. An airway assessment was also conducted using Mallampati grading to predict intubation ease based on the visibility of oropharyngeal structures. After the study procedures and potential complications were explained, written informed consent was obtained from each participant.

During enrollment, patients were introduced to the Satisfaction VAS, a 10-cm scale used to measure their satisfaction level - where a score of 0 represents “extremely unsatisfied” and 10 indicates “extremely satisfied.”

Procedures

Following randomization into either the ERAS protocol (EP) group or the routine protocol (RP) group in a 1:1 ratio, patients were managed according to their assigned protocol. Upon transfer to the preoperative holding area, all patients were fitted with standard monitoring devices, including a pulse oximeter, non-invasive blood pressure monitor, and ECG leads. Baseline measurements of heart rate, blood pressure, and oxygen saturation were recorded, and a 20-gauge intravenous cannula was secured for the administration of fluids and medications.

Comparison of RP and EP

In the RP group, patients did not receive any advance perioperative education, and they followed a fasting period of six hours before surgery. The anesthetic protocol included bupivacaine heavy 0.5% (10 mg) with buprenorphine (60 mcg). For postoperative pain management, intravenous paracetamol (1 g) was administered, followed by oral paracetamol (650 mg) for the next two days. Breakthrough pain was managed with intravenous tramadol hydrochloride (100 mg) when the VAS score exceeded 3 or if the patient requested additional analgesia. To prevent postoperative nausea and vomiting, ondansetron (4 mg) was administered intravenously 30 minutes before surgery. Standard intraoperative uterotonics included oxytocin (5 IU) slow intravenous bolus, followed by 10 IU diluted in 250 ml normal saline. Prophylactic antibiotics, including ceftriaxone (1 g) and metronidazole (500 mg), were given intravenously 30-60 minutes before the incision. Crystalloid infusion was used to manage hypotension, with a phenylephrine (100 mcg) bolus administered if required. Intraoperative warming measures were not actively implemented. Postoperatively, oral intake was resumed only after the return of bowel movements, and mobilization was initiated based on the patient’s preference. Skin-to-skin breastfeeding was left to the patient’s discretion.

In the EP group, patients received detailed perioperative education, covering pain management strategies, the importance of early feeding, mobilization, lactation support, discharge criteria, and follow-up care. They were allowed clear fluid intake up to two hours before surgery. The anesthetic protocol was the same as in the RP group, with bupivacaine heavy 0.5% (10 mg) and buprenorphine (60 mcg). Postoperative pain management was enhanced with an ultrasound-guided bilateral TAP block, using 20 ml of ropivacaine 0.5% and 4 mg of dexamethasone [9] administered on each side after skin closure, followed by oral paracetamol (650 mg) for the next two days. Breakthrough pain was managed similarly with intravenous tramadol hydrochloride (100 mg) if the VAS score exceeded 3 or if the patient requested additional analgesia. Granisetron (3 mg) was administered intravenously before the incision to prevent postoperative nausea and vomiting [10]. Intraoperative uterotonics and prophylactic antibiotics were identical to those used in the RP group. However, perioperative fluid and blood pressure management in the ERAS group emphasized avoiding overhydration and using a combination of fluids and vasopressors to counteract hypotension. Spinal anesthesia was administered after prophylactic phenylephrine (100 mcg). Intraoperative warming was actively managed using a warm air blower, with the patient’s bed pre-warmed before surgery. Postoperatively, oral intake was initiated within two hours of surgery, starting with a light meal of less than 200 ml and gradually increasing as tolerated. Mobilization was initiated six hours after the time of spinal anesthesia. Early skin-to-skin contact with the newborn was encouraged immediately upon return to the ward, and breastfeeding was initiated within the first hour after surgery.

Statistical analysis

Statistical analyses were performed using IBM SPSS Statistics for Windows, Version 20.0 (Released 2011; IBM Corp., Armonk, NY, USA). The independent t-test was used to compare the means of two groups when the data were parametric (normally distributed). The Mann-Whitney U test was used as the nonparametric equivalent of the independent t-test. Fisher’s exact test was applied for categorical variables when expected cell counts were small; otherwise, Pearson’s chi-squared test was used. Quantitative data were presented as mean ± SD, while categorical data were presented as proportions. The quantitative data were analyzed using the independent t-test, and categorical data were analyzed using the chi-squared test or Fisher’s exact test, as appropriate. Statistical significance was set at p < 0.05 for all analyses.

## Results

Age distribution

**Figure 1:**
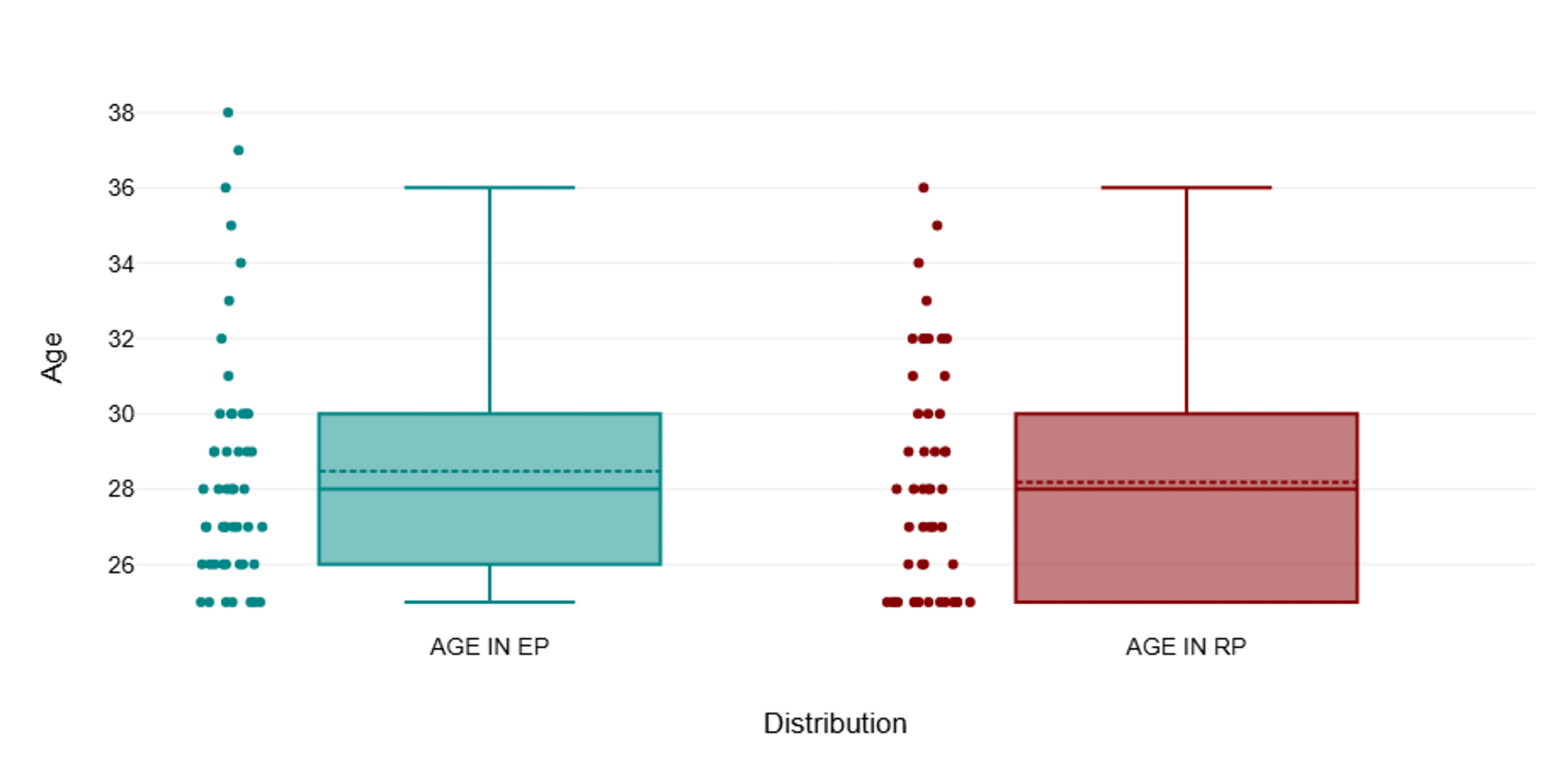
Mean and SD of age among groups EP, enhanced recovery after surgery protocol; RP, routine protocol

A total of 100 patients were enrolled in the study, with comparable age distributions between the two groups, showing no significant difference (p = 0.633). The majority of participants in both the EP and RP groups were aged between 25 and 27 years, with 24 patients in each group. In the 28-31 age range, there were 19 patients in the EP group and 16 in the RP group. A slightly higher number of participants aged 32-35 were in the RP group (nine) compared to the EP group (four). Conversely, the EP group had more participants in the 36-39 age range (three) compared to the RP group (one), as shown in Figure 1. Overall, the age distribution was well-balanced across both groups, as presented in Table 1 and Table 2.

**Table 1:** Age (in years) distribution between the groups Statistical test used: independent t-test EP, enhanced recovery after surgery protocol; RP, routine protocol

Age (in years)	Group EP (N = 50)	Group RP (N = 50)	p-Value
25-27	24	24	0.633
28-31	19	16	
32-35	4	9	
36-39	3	1	

**Table 2:** Mean and SD of age (in years) between the groups EP, enhanced recovery after surgery protocol; RP, routine protocol

Statistic	Group EP	Group RP
Mean	28.48	28.18
SD	3.2023	3.0619
Maximum	38	36
Minimum	25	25

Parity distribution

The parity distribution between the two groups showed no statistically significant difference (p = 0.591). Sixteen participants had a parity of 1, with seven in the EP group and nine in the RP group. The majority had a parity of 2 (41 patients), with a slightly higher number in the RP group (22) compared to the EP group (19). For participants with three or more deliveries, there were more in the EP group (24) than in the RP group (19). Overall, the parity distribution was balanced across both groups, as shown in Table 3.

**Table 3:** Parity and gestation distribution between the groups Statistical test used: Pearson’s chi-squared test EP, enhanced recovery after surgery protocol; RP, routine protocol

Category	Variable	Group EP (N = 50)	Group RP (N = 50)	Total (N = 100)	% within EP	% within RP	p-Value
Parity	1	7	9	16	43.80%	56.30%	0.591
	2	19	22	41	46.30%	53.70%	
	3 or more	24	19	43	55.80%	44.20%	
Gestation	Total	50	50	100	50.00%	50.00%	0.558
	Single	48	49	97	49.50%	50.50%	
	Twin	2	1	3	66.70%	33.30%	
	Total	50	50	100	50.00%	50.00%	

Intraoperative vomiting and shivering

The incidence of intraoperative vomiting was low and did not differ significantly between the two groups (p = 0.315). Almost all patients (99 out of 100) did not experience vomiting, with 49 in the EP group and 50 in the RP group. Only one patient in the EP group experienced intraoperative vomiting, while no cases were reported in the RP group. This suggests that intraoperative vomiting was not a major issue in either group. On the other hand, intraoperative shivering was significantly more common in the RP group than in the EP group (p = 0.004). A total of 22 patients experienced shivering, with 17 in the RP group and 5 in the EP group. Among those who did not experience shivering, 45 were in the EP group, compared to 33 in the RP group, as shown in Table 4.

**Table 4:** Intraoperative vomiting and shivering distribution between the groups Statistical test used: Pearson’s chi-squared test EP, enhanced recovery after surgery protocol; RP, routine protocol

Category	Variable	Group EP (N = 50)	Group RP (N = 50)	Total (N = 100)	% within EP	% within RP	p-Value
Intraoperative vomiting	No	49	50	99	49.50%	50.50%	0.315
	Yes	1	0	1	100.00%	0.00%	
	Total	50	50	100	50.00%	50.00%	
Intraoperative shivering	No	45	33	78	57.70%	42.30%	0.004
	Yes	5	17	22	22.70%	77.30%	
	Total	50	50	100	50.00%	50.00%	

Intraoperative hypotension

In Group EP, 10 out of 50 patients experienced intraoperative hypotension. In contrast, 32 out of 50 patients in Group RP had intraoperative hypotension. The incidence of hypotension was higher in Group RP compared to Group EP. The p-value is less than 0.05, indicating a significant difference in the occurrence of intraoperative hypotension between the two groups, as shown in Table 5.

**Table 5:** Intraoperative hypotension between the groups Statistical test used: Pearson’s chi-squared test EP, enhanced recovery after surgery protocol; RP, routine protocol

Intraoperative hypotension	EP	RP	Total	p-Value
No	40	18	58	0.000
% within category	69.00%	31.00%	100%	
Yes	10	32	42	
% within category	23.80%	76.20%	100%	
Total	50	50	100	

Intraoperative nausea

In Group EP, five out of 50 patients experienced intraoperative nausea. In Group RP, six out of 50 patients had nausea intraoperatively. The p-value was greater than 0.05, indicating no significant difference in the occurrence of intraoperative nausea between the two groups, as shown in Table 6.

**Table 6:** Intraoperative nausea Statistical test used: Pearson’s chi-squared test EP, enhanced recovery after surgery protocol; RP, routine protocol

Intraoperative nausea	EP	RP	Total	p-Value
No	45	44	89	0.749
% within category	50.60%	49.40%	100.00%	
Yes	5	6	11	
% within category	45.50%	54.50%	100.00%	
Total	50	50	100	

Postoperative nausea

In Group EP, nine out of 50 patients experienced postoperative nausea. In Group RP, 17 out of 50 patients had nausea postoperatively. The p-value was 0.068, suggesting no statistically significant difference in postoperative nausea between the two groups, as shown in Table 7.

**Table 7:** Postoperative nausea Statistical test used: Pearson’s chi-squared test EP, enhanced recovery after surgery protocol; RP, routine protocol

Postoperative nausea	EP	RP	Total	p-Value
No	41	33	74	0.068
% within category	55.40%	44.60%	100.00%	
Yes	9	17	26	
% within category	34.60%	65.40%	100.00%	
Total	50	50	100	

Postoperative vomiting

In Group EP, three out of 50 patients experienced postoperative vomiting. In Group RP, seven out of 50 patients had vomiting postoperatively. The p-value was >0.05, indicating no significant difference in the occurrence of postoperative vomiting between the two groups, as shown in Table 8.

**Table 8:** Postoperative vomiting Statistical test used: Pearson’s chi-squared test EP, enhanced recovery after surgery protocol; RP, routine protocol

Postoperative vomiting	EP	RP	Total	p-Value
No	47	43	90	0.182
% within category	52.20%	47.80%	100.00%	
Yes	3	7	10	
% within category	30.00%	70.00%	100.00%	
Total	50	50	100	

Postoperative pain assessment

**Figure 2:**
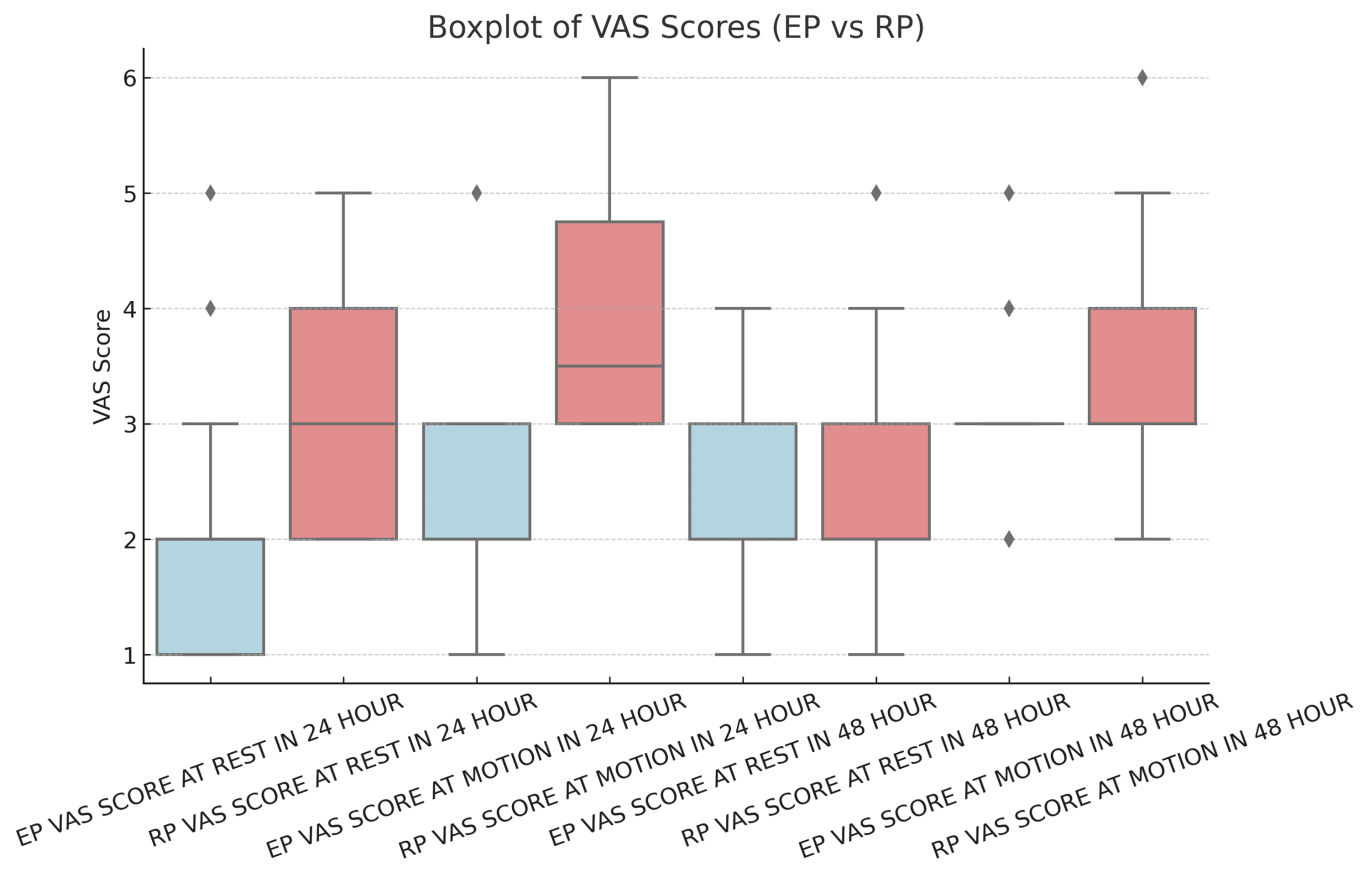
Boxplot of VAS scores (EP vs. RP) The box plot displays the distribution of VAS scores for both the EP and RP groups under different conditions. Each box represents the IQR, which contains the middle 50% of the data, and the median is marked inside the box. EP, enhanced recovery after surgery protocol; RP, routine protocol; VAS, Visual Analogue Scale

**Figure 3:**
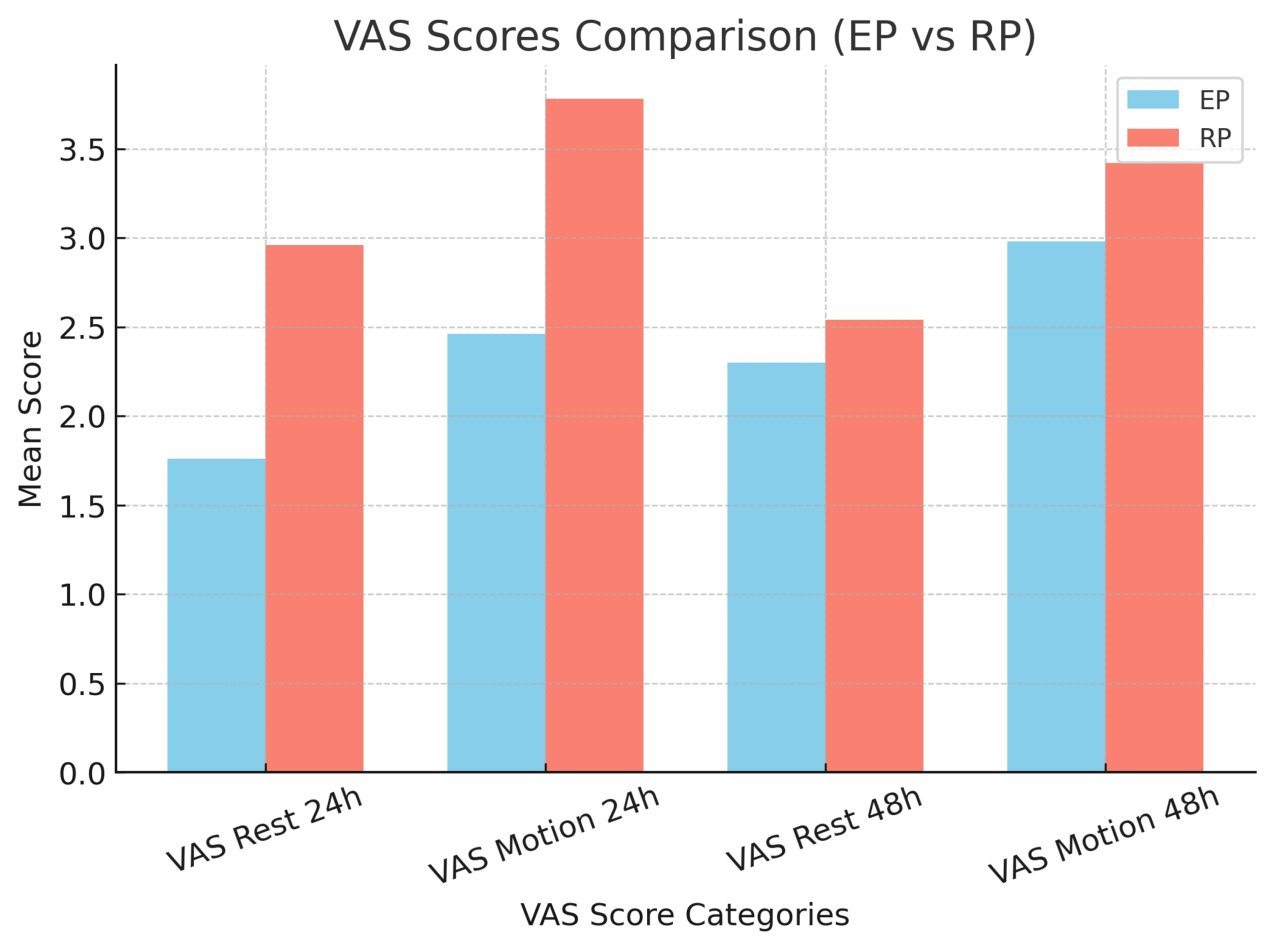
VAS score at various time intervals between the groups EP, enhanced recovery after surgery protocol; RP, routine protocol; VAS, Visual Analogue Scale

Postoperative pain was assessed using the VAS at 24 hours post-surgery. At rest, patients in the EP group had a significantly lower mean VAS score (1.76 ± 0.82) compared to the RP group (2.96 ± 0.95) (p < 0.05). When assessed during motion at 24 hours post-surgery, the mean VAS score in Group EP (2.46 ± 0.81) was significantly lower than that in Group RP (3.78 ± 0.89) (p < 0.05). However, at 48 hours post-surgery, the mean VAS score at rest in Group EP (2.3 ± 0.86) was comparable to that in Group RP (2.54 ± 0.93) (p > 0.05). Similarly, for pain during motion at 48 hours post-surgery, the mean VAS score in Group EP (2.98 ± 0.71) was comparable to that in Group RP (3.42 ± 0.95) (p > 0.05), as shown in Table 9, Figure 2, and Figure 3.

**Table 9:** Postoperative pain assessment using VAS Statistical test used: independent t-test EP, enhanced recovery after surgery protocol; RP, routine protocol; VAS, Visual Analogue Scale

Characteristics of VAS	Group EP (N = 50)	Group RP (N = 50)	p-Value
Rest in 24 hours	1.76 ± 0.8221	2.96 ± 0.9467	0.000
Motion in 24 hours	2.46 ± 0.8134	3.78 ± 0.8873	0.000
Rest in 48 hours	2.3 ± 0.8631	2.54 ± 0.9304	0.184
Motion 48 hours	2.98 ± 0.714	3.42 ± 0.9495	0.010

Opioid consumption

The number of patients requiring opioids at 24 hours post-surgery was significantly lower in Group EP (two patients, 4%) compared to Group RP (25 patients, 50%), with a p-value of less than 0.05. At 48 hours, the number of patients requiring opioids in Group EP (eight patients, 16%) and Group RP (19 patients, 38%) was comparable, with a p-value greater than 0.05. Furthermore, the need for extra analgesics was significantly lower in Group EP (nine patients, 18%) compared to Group RP (32 patients, 64%), with a p-value of less than 0.05, as shown in Table 10.

**Table 10:** Opioid consumption between the groups Statistical test used: Pearson’s chi-squared test EP, enhanced recovery after surgery protocol; RP, routine protocol

Characteristics	Group EP (N = 50)	Group RP (N = 50)	p-Value
Number of patients required opioids at 24 hours	2 (4%)	25 (50%)	0.000
Number of patients required opioids at 48 hours	8 (16%)	19 (38%)	0.013
Requirement of extra analgesics	9 (18%)	32 (64%)	0.000

Satisfaction VAS, length of stay, and average cost of hospitalization

The satisfaction VAS in Group EP (6.18 ± 0.90) is significantly higher than in Group RP (4.76 ± 0.62), with a p-value of less than 0.05. The total length of stay in the hospital for Group EP (3.76 ± 0.80 days) is significantly shorter than for Group RP (4.68 ± 0.71 days), with a p-value of less than 0.05. The postoperative length of stay in the hospital for Group EP (3.04 ± 0.73 days) is significantly shorter than for Group RP (3.94 ± 0.71 days), with a p-value of less than 0.05. The average cost of hospitalization in rupees for Group EP (6606 ± 925.43) and Group RP (7100 ± 852.16) differs significantly, with a p-value of 0.007, as shown in Table 11.

**Table 11:** Satisfaction VAS, length of stay, and average cost of hospitalization Statistical test used: independent t-test EP, enhanced recovery after surgery protocol; RP, routine protocol; VAS, Visual Analogue Scale

Characteristics	Group EP (N = 50)	Group RP (N = 50)	p-Value
Satisfaction VAS	6.18 ± 0.8965	4.76 ± 0.6247	0.000
Total length of hospital stay in days	3.76 ± 0.7969	4.68 ± 0.7126	0.000
Postoperative length of stay in days	3.04 ± 0.7273	3.94 ± 0.7117	0.000
Average cost of hospitalization in rupees	6,606 ± 925.4299	7100 ± 852.1617	0.007

## Discussion

Giving birth is a profound joy for women. However, for those who choose cesarean delivery - whether by choice or due to maternal and fetal conditions - the initial happiness is often short-lived due to postoperative challenges. Pain, sedation, and complications such as shivering, nausea, and vomiting can prevent mothers from fully engaging with their newborns, hindering activities like holding and feeding. This not only concerns the mother but also impacts the well-being of the newborn. To address the limitations of traditional protocols in cesarean delivery, we aimed to study the implementation of ERAS protocols.

Numerous studies on ERAS have demonstrated promising results, particularly in elective colonic or rectal resection [11], emergency laparotomy [12], and benign gynecological surgeries [13]. Inadequate management of acute pain in surgical patients leads to several negative outcomes, including increased morbidity, reduced physical function and quality of life, delayed recovery, prolonged opioid use, and higher healthcare costs [14]. In our study, patients in the EP group received bilateral TAP block, utilizing 20 ml of 0.5% ropivacaine and 1 ml of dexamethasone, administered as 21 ml on each side, totaling 42 ml, following a cesarean section under spinal anesthesia. Our findings showed that a TAP block, using 42 mL of 0.5% ropivacaine and 1 ml of dexamethasone, significantly reduced pain intensity and analgesic consumption over a 24-hour postoperative period. Better postoperative analgesia contributed to earlier patient mobilization. Similarly, Ripollés et al. conducted a multicenter review study showing that TAP blocks decrease the requirement for analgesia and lower VAS scores within the first 24 hours postoperatively. Our study supports this, showing that TAP blocks effectively reduce opioid consumption and lower VAS scores following cesarean section [15].

Regarding intraoperative hypotension, patients in the EP group received prophylactic intermittent boluses of phenylephrine (100 mcg), which was compared with the RP group. A prophylactic phenylephrine bolus effectively prevented post-spinal anesthesia hypotension in 80% of patients during cesarean section, consistent with findings from Guo et al. [16]. Additionally, patients in the EP group were actively warmed using a forced air warmer and passively by using a fluid warmer, which was compared with the RP group. The combined use of a forced air warmer and a fluid warmer effectively reduced intraoperative shivering in the EP group [17].

Patient satisfaction was also assessed using a VAS at discharge. Group EP showed significantly higher satisfaction, with a mean score of 6.18 compared to 4.76 in Group RP. This result aligns with Karki and Saha [18], who evaluated patient satisfaction with the ERAS protocol in elective cesarean sections - the most common surgical procedure globally. Most women in their study were satisfied with the surgical experience and would opt for the same protocol in the future.

In our study, patients in the EP group had a significantly shorter total length of stay (3.76 days) and postoperative length of stay (3.04 days) compared to the RP group, which had a total length of stay (4.68 days) and postoperative length of stay (3.94 days). These results align with findings from Mullman et al. [19]. Additionally, there was a significant reduction in hospitalization costs for Group EP (₹6,606 ± 925.43) compared to Group RP (₹7,100 ± 852.16), with a p-value of 0.007. This difference is likely due to the tailored ERAS protocol, which enhanced recovery, reduced complications, and enabled earlier discharge. Nonetheless, the study’s single-center design, lack of long-term follow-up on opioid use and maternal functional recovery, and absence of a cost-effectiveness analysis may limit its potential for broader policy recommendations.

## Conclusions

The implementation of the tailored ERAS protocol for acute pain management in cesarean deliveries led to more effective pain control, fewer intraoperative complications such as hypotension and shivering, reduced opioid consumption, earlier mobilization, shorter hospital stays, lower overall healthcare costs, and higher patient satisfaction. These findings highlight the benefits of incorporating the tailored ERAS protocol into standard cesarean delivery care to improve postoperative pain management and enhance overall perioperative outcomes.

## References

[1] Dahlgren G, Hultstrand C, Jakobsson J, Norman M, Eriksson EW, Martin H (1997). Intrathecal sufentanil, fentanyl, or placebo added to bupivacaine for cesarean section. Anesth Analg.

[2] Schoenwald A, Clark CR (2006). Acute pain in surgical patients. Contemp Nurse.

[3] Demelash G, Berhe YW, Gebregzi AH (2006). Prevalence and factors associated with postoperative pain after cesarean section at a comprehensive specialized hospital in Northwest Ethiopia: prospective observational study. Open Access Surgery.

[4] Wilson RD, Caughey AB, Wood SL (2018). Guidelines for antenatal and preoperative care in cesarean delivery: enhanced recovery after surgery society recommendations (part 1). Am J Obstet Gynecol.

[5] Caughey AB, Wood SL, Macones GA (2018). Guidelines for intraoperative care in cesarean delivery: enhanced recovery after surgery society recommendations (part 2). Am J Obstet Gynecol.

[6] Macones GA, Caughey AB, Wood SL (2019). Guidelines for postoperative care in cesarean delivery: Enhanced Recovery After Surgery (ERAS) society recommendations (part 3). Am J Obstet Gynecol.

[7] McDonnell JG, Curley G, Carney J, Benton A, Costello J, Maharaj CH, Laffey JG (2008). The analgesic efficacy of transversus abdominis plane block after cesarean delivery: a randomized controlled trial. Anesth Analg.

[8] Pan J, Hei Z, Li L (2020). The advantage of implementation of enhanced recovery after surgery (ERAS) in acute pain management during elective cesarean delivery: a prospective randomized controlled trial. Ther Clin Risk Manag.

[9] Zufferey PJ, Chaux R, Lachaud PA, Capdevila X, Lanoiselée J, Ollier E (2024). Dose-response relationships of intravenous and perineural dexamethasone as adjuvants to peripheral nerve blocks: a systematic review and model-based network meta-analysis. Br J Anaesth.

[10] Parripati LD, Bandarupalli SS, Ramella M, Sandhya L (2021). A comparison between intravenous ondansetron and granisetron in the preventive management of postoperative nausea and vomiting in patients undergoing cesarean section under spinal anesthesia. Bali J Anesthesiol.

[11] Teeuwen PH, Bleichrodt RP, Strik C (2010). Enhanced recovery after surgery (ERAS) versus conventional postoperative care in colorectal surgery. J Gastrointest Surg.

[12] Purushothaman V, Priyadarshini P, Bagaria D (2021). Enhanced recovery after surgery (ERAS) in patients undergoing emergency laparotomy after trauma: a prospective, randomized controlled trial. Trauma Surg Acute Care Open.

[13] Bahadur A, Kumari P, Mundhra R (2021). Evaluate the effectiveness of enhanced recovery after surgery versus conventional approach in benign gynecological surgeries: a randomized controlled trial. Cureus.

[14] Gan TJ (2017). Poorly controlled postoperative pain: prevalence, consequences, and prevention. J Pain Res.

[15] Ripollés J, Mezquita SM, Abad A, Calvo J (2015). Analgesic efficacy of the ultrasound-guided blockade of the transversus abdominis plane - a systematic review. Braz J Anesthesiol.

[16] Guo L, Xu X, Qin R (2023). Prophylactic norepinephrine and phenylephrine boluses to prevent postspinal anesthesia hypotension during cesarean section: a randomized sequential allocation dose-finding study. Drug Des Devel Ther.

[17] Jun JH, Chung MH, Jun IJ (2019). Efficacy of forced-air warming and warmed intravenous fluid for prevention of hypothermia and shivering during caesarean delivery under spinal anaesthesia: a randomised controlled trial. Eur J Anaesthesiol.

[18] Karki D, Saha R (2021). Assessment of patient satisfaction after implementing an enhanced recovery after surgery (ERAS) protocol for elective caesarean sections. J Kathmandu Medical College.

[19] Mullman L, Hilden P, Goral J (2020). Improved outcomes with an enhanced recovery approach to cesarean delivery. Obstet Gynecol.

